# Detection of Epistatic and Gene-Environment Interactions Underlying Three Quality Traits in Rice Using High-Throughput Genome-Wide Data

**DOI:** 10.1155/2015/135782

**Published:** 2015-08-04

**Authors:** Haiming Xu, Beibei Jiang, Yujie Cao, Yingxin Zhang, Xiaodeng Zhan, Xihong Shen, Shihua Cheng, Xiangyang Lou, Liyong Cao

**Affiliations:** ^1^Institute of Crop Science and Institute of Bioinformatics, College of Agriculture and Biotechnology, Zhejiang University, Hangzhou 310058, China; ^2^State Key Laboratory of Rice Biology and Zhejiang Key Laboratory of Super Rice Research, China National Rice Research Institute, Hangzhou 311401, China; ^3^Department of Biostatistics, University of Alabama at Birmingham, Birmingham, AL 35294, USA

## Abstract

With development of sequencing technology, dense single nucleotide polymorphisms (SNPs) have been available, enabling uncovering genetic architecture of complex traits by genome-wide association study (GWAS). However, the current GWAS strategy usually ignores epistatic and gene-environment interactions due to absence of appropriate methodology and heavy computational burden. This study proposed a new GWAS strategy by combining the graphics processing unit- (GPU-) based generalized multifactor dimensionality reduction (GMDR) algorithm with mixed linear model approach. The reliability and efficiency of the analytical methods were verified through Monte Carlo simulations, suggesting that a population size of nearly 150 recombinant inbred lines (RILs) had a reasonable resolution for the scenarios considered. Further, a GWAS was conducted with the above two-step strategy to investigate the additive, epistatic, and gene-environment associations between 701,867 SNPs and three important quality traits, gelatinization temperature, amylose content, and gel consistency, in a RIL population with 138 individuals derived from super-hybrid rice Xieyou9308 in two environments. Four significant SNPs were identified with additive, epistatic, and gene-environment interaction effects. Our study showed that the mixed linear model approach combining with the GPU-based GMDR algorithm is a feasible strategy for implementing GWAS to uncover genetic architecture of crop complex traits.

## 1. Introduction

Rice (*Oryza sativa* L.), a crop species of economic importance, provides the staple food for more than half of the population in the world. In China, the super-hybrid rice plays a pivotal role in the country's food security. There are almost eighty super-rice varieties, such as Xieyou9308, that have been successfully bred and commercially released to rice farmers since the super-rice breeding program was initiated by the Chinese government in 1996 [1]. Substantial geneticist's and breeder's effort is being expended in attempt to further investigate the mechanisms underlying high yield potential, wide adaptability, better grain quality, better disease resistance, and strong resistance to lodging in super-hybrid rice. The majority of these traits are quantitatively inherited. In addition to the increase of grain yield and the improvement of living conditions, more attention has been being paid toward improving grain quality, related to preference of cooking and eating quality of rice varieties.

As an important grain quality trait for rice, the level of amylose content (AC) is positively correlated with resistant starch (RS) content of granular starches [2–10], which is defined as the portion of dietary starch that is not digested in the small intestine of a healthy human [11]. Gelatinization temperature (GT), the critical temperature at which about 90% of the starch granules have swelled irreversibly in hot water and start to lose crystallinity and birefringence, is another important criterion for rice quality related to cooking quality [12]. Gel consistency (GC), a measure of cold paste-viscosity of cooked milled rice flour, is a good index of cooked rice texture, especially for rice with high AC. Breeders are trying to develop high-yielding varieties with soft GC [12] because rices with soft GC cook tender and remain soft even upon cooling [13]. Therefore, understanding the genetic basis of these key traits associated with grain quality is essential to predictive rice improvement.

As high-throughput technologies producing dense single nucleotide polymorphisms (SNPs) across the whole genome, the genome-wide association study (GWAS) provides us with insightful information into genetic architecture of complex traits and is a common approach to uncover genetic components of agronomic traits. Association mapping is a high-resolution method to map quantitative trait SNPs (QTSs) based on linkage disequilibrium (LD). Association analytical methods can evaluate whether certain alleles within a population are correlated with the phenotypes of interest more frequently than the expected ones under the null hypothesis. Thus, the limitations in the traditional linkage mapping due to the statistical ambiguity with insufficient molecular markers can be alleviated. It has been widely applied in plant resource populations such as rice, maize, barley, and wheat recently [14–19].

However, the current GWAS analysis fails to detect epistatic and gene-environment interactions in most studies such as maize. But phenotypes of all living organisms represent the consequence of several genetic components including epistatic effects and their interactions with environment; therefore, to estimate genetic merit relevant to the epistases and their interactions with environment certainly plays a crucial role in planning an effective breeding regime [20, 21]. Searching for only main effects may miss the key genetic variants with specific environment response in the context of complex traits and it is not likely to provide reliable estimates of genetic component effects [22].

On the other hand, due to the prohibitively intensive computation required for a GWAS, the available methods are unpractical and difficult to extend for detection of gene-gene, gene-environment, and gene-gene-environment interactions in an experimental data with multiple environments with enormous SNPs. Currently, a workable solution is provided with the availability of generalized multifactor dimensionality reduction (GMDR) algorithm on a computing system with graphics processing units (GPUs), a type of hardware implementation of parallel computation that can be adapted for many scientific tasks [23]. The present study first used the GMDR-GPU software to screen potential candidate variants and then used the mixed liner model to dissect the epistatic and gene-environmental interactions of GT, AC, and GC in a super-hybrid rice Xieyou9308 derived RIL population, which was preferentially selected as the cardinal population for the development of BCF1 populations or immortalized F2 populations for the identification of QTLs associated with important agronomic traits [24–26]. Before analyzing the real data, Monte Carlo simulations were carried out to test the reliability and efficiency of the model and methods.

## 2. Materials and Methods

### 2.1. Field Experiment and DNA Resequencing for Genotyping

A RIL mapping population consisting of 138 lines, derived from super-hybrid rice Xieyou9308, was planted in Linshui City, Hainan Province, and Hangzhou City, Zhejiang Province, in 2009, respectively. Three quality traits, GT, AC, and GC, were investigated.

DNA resequencing was conducted in Beijing Genomics Institute (BGI) for two parents with 10X coverage and 138 lines with 2X coverage. The latest version of Nipponbare sequence [27] was used as the reference genome. Sequence alignment was conducted between the sequencing sequence and the reference genome with the software of Burrows-Wheeler Aligner (BWA) [28]. SNPs were searched between the individuals and the reference genome using the software of Sequence Alignment/Map Tools (SAMtools) [29] with the criteria of base quality over 30, mapping quality over 20, and the maximum sequence depth less than 1000. All the results were integrated by Perl program. A total of 701,867 SNPs were generated from DNA resequencing for the subsequent association study.

### 2.2. Genetic Models and Statistical Methods

Association mapping was performed using the mixed linear model approach. Suppose that the genetic variation of one quantitative trait is controlled by *s* genes. An experiment under multiple environments is conducted for gene mapping. The phenotypic value of the *k*th individual in the *h*th environment (*y*_*hk*_) can be expressed by the following mixed linear model:

(1) yhk=μ+∑i=1sxikai+∑i<jxikxjkaaij+eh+∑ixikaehi+∑i<jxikxjkaaehij+εhk,

where *μ* is the population mean; *a*_*i*_ is the additive effect of the *i*th SNP with coefficient *x*_*ik*_, fixed effect; *aa*_*ij*_ is the additive × additive interaction effect between the *i*th SNP and the *j*th SNP with coefficient *x*_*ik*_ *x*_*jk*_, fixed effect; *e*_*h*_ is the random effect of the *h*th environment; *ae*_*hi*_ is the additive × environment interaction effect of the *i*th SNP and the *h*th environment effect with coefficient *x*_*ik*_, random effect; *aae*_*hij*_ is the interaction effect of *aa*_*ij*_ with the *h*th environment with coefficient *x*_*ik*_ *x*_*jk*_; and *ε*_*hk*_ is the random residual effect of the *k*th individual in the *h*th environment. The coefficient *x*_*ik*_ can be determined according to the genotype of the SNP, taking values of 1 and −1 for the homozygotes of high and low frequency alleles, respectively, and of 0 for the heterozygote.

A two-step mapping strategy for GWAS was employed to dissect genetic architecture of AC, GC, and GT. First, we used GMDR method [30] to screen SNPs potentially associated with phenotype using 1-locus model, 2-locus model, and 3-locus model, respectively; a set of reduced number of candidate SNPs was obtained for the AC, GC, and GT. Based on these potential SNPs, the model (1) was applied for significance test of the genetic effects due to individual SNP and paired SNPs in terms of *F*-statistic and the threshold specified by the permutation method at the experiment-wise error rate of 0.05. Then, all the significant SNPs were used to build a full model and the MCMC method was employed to generate the distribution of each effect in the full model. On the basis of the distribution, each effect was estimated by the mean and its significance is tested by *t*-statistic [31]. The data analysis by the two-step strategy was implemented with a newly developed GWAS software called QTXNetwork (http://ibi.zju.edu.cn/software/QTXNetwork/).

## 3. Results

### 3.1. Monte Carlo Simulations

To investigate the efficiency and accuracy of the proposed methods, we performed a series of Monte Carlo simulations to verify the unbiasedness and robustness as well as statistical power. Because our real experiment data is based on a rice RIL population, we conducted the simulations based on this kind of population to examine our methods. Two environments were considered. 525 SNPs were scattered across 3 chromosomes, with 175 markers evenly distributed on each chromosome. Three individual QTSs controlling the quantitative trait were assigned on 3 chromosomes and two-paired epistatic QTSs were also set with gene-gene-environment interaction effects. Our simulations would also investigate the influences of heritability and population size on estimation of QTS parameters. Different heritabilities and population sizes were used in simulations. Each simulation generated the experimental samples according to the parameter setting for analysis and the results from 200 simulations are summarized in Tables 1–4.

Under a heritability of 70%, we conducted simulations to investigate the effectiveness and robustness of our proposed method in estimating QTS parameters, according to the results in Tables 1 and 2, it was clear that all the QTSs could be detected, and all kinds of effects could be estimated effectively under the three population sizes. Most estimates were close to true values of parameter, and the estimation accuracy of QTS effects was acceptable, especially for the QTS main effects. The estimates of additive-additive interaction effects with environments were less accurate, which might be due to the interference additive-additive effects. The statistical power increased as the population size became bigger and most of the power under the population size over 150 individuals was 100% except SNP.93 on the third chromosome.

In order to understand the robustness of our method, we conducted simulations under two heritabilities of 70% and 50%, respectively, under the population size of 150 individuals. The simulation results (Tables 3 and 4) clearly showed a very accurate detection of QTSs and estimation of QTS effects. All the estimates of QTS positions and effects were quite robust for different heritabilities, but the epistatic interaction effects with environments between SNP.44 on the second chromosome and SNP.63 on the third chromosome deviated a little from the true value. Additionally, the power did not changed very much when the heritability was increased from 50% to 70%.

In conclusion, according to the increase of population size, the power values of QTS with median effects became higher and the estimated effects were closer to the true parameter values. Therefore, a population consisting of around 150 or more RILs is a reasonable size to maintain the estimation efficiency and power for a trait controlled by modest QTS effects.

### 3.2. The Genetic Architecture of Rice Quality Traits

Based on the above simulation consequences, an association study was conducted to analyze the genetic architecture for three quality traits of super-hybrid rice Xieyou9308 RIL population with 138 lines in two environments and the results were listed in Table 5. Totally, four QTSs were detected on all the rice chromosomes for the three quality traits, 2 QTSs for AC, 2 QTSs for GC, and 2 QTSs for GT, respectively. One QTS seemed to be pleiotropic for all the three traits. The total heritability estimated by the full model for the three traits was all over 50%, of which the highest was 68.67% for AC and the lowest was 52.01% for GT.

For AC, two QTSs were detected and involved only in additive effects with very high significance and both of effects were negative. Within them, the heritability of rs1644460 was as high as 51.78% whose proportion was up to 75.4% of total heritability.

There were also two QTSs discovered for GC. Positive additive effect of rs1644460 and negative additive effect of rs919289 were detected, similar to AC; the former, with an extremely high significance, accounted for more than 89% of the genetic variance of GC. The special QTS, rs1644460, was significant in both environments but showed varying effects in different environments, indicating an unstable expression of this QTS across different environments.

For GT, two significant QTSs were identified, which were involved not only in additive effects and additive × environment interaction effects but also in epistasis and interaction of epistasis with environments. The QTS, rs1289107, with highest additive effect reached the highest heritability (24%) of all the effects. Similar to GC, there were two QTSs, rs1289107 and rs1644460, which were expressed in distinct patterns under different environments and they reached high heritability in interaction effects of additive with environments, indicating that the expression of these two QTSs depended substantially on environments. It should be noted that one-paired epistatic QTSs were detected for GT with different pattern epistasis × environment interaction effects.

Throughout the three traits, rs1644460 was detected for all the three quality traits with high significance and heritability, suggesting a pleiotropic role of the QTS for the three traits. Generally, there were diverse patterns in genetic effects of QTSs among three quality traits. The QTSs controlling all the three traits expressed mainly in genetic main effects and subtle environment-specific additive or additive-additive epistasis effects were detected. Furthermore, only two highly significant QTSs (rs1644460 and rs1289107) were expressed both in genetic main effects and genetic-environment interaction effects. On the contrary, two QTSs (rs1610021 and rs919289) were expressed mainly in additive effects and modestly in genetic-environment interaction effects for AC and GC. Conclusively, environment is a very crucial factor to affect gene expression for rice quality traits. Most QTSs interact with environment and the environment can enhance or weaken the expression of most QTSs for the three quality traits.

## 4. Discussion

Epistasis and its interaction with environment have been recognized as important components of cultivar performance and have received more attention in rice breeding programs. Nevertheless, these effects have not yet effectively been analyzed by the current GWASs for the reason of absence of appropriate statistical methodology and heavy burden of computation [32–34]. If a reduced model ignoring epistasis and gene-environment interaction is employed, the resulting GWAS would give biased estimation of effects, poor detection precision and power, and low heritability to explain variation of complex traits [34–37]. This study used a QTS full model including the additive effect, the additive-additive epistatic effect, and their interaction effects with multienvironments of each QTS, to analyze genetic architecture of gelatinization temperature, amylose content, and gel consistency so that the estimation accuracy of each effect will be greatly improved benefiting from elimination of false positive QTS by permutation method and more accurate estimation of residual effect. In order to alleviate the computing cost, we first used the GMDR algorithm on GPU to screen the potential associated markers and then conducted one-dimensional and two-dimensional searching to detect putative QTSs with the screened markers as cofactors to control background genetic effects. Totally, we identified four QTSs with additive effects, epistasis, and environment interaction effects for the three quality traits.

Traditional plant breeding is based on phenotypic selection of superior genotypes among segregating progenies, and its effectiveness is often affected by environment and genotype-environment interaction. Therefore, it sometimes leads to unreliable selection of some traits [38–40]. Although marker-assisted selection (MAS) is an effective way to improve the efficiency and precision of plant breeding, it is still under the influence of the strength in association between markers and genes of target traits. As the highly significant SNPs identified by GWAS mapping mostly link tightly to the genes controlling target traits, with assistance of these detected QTSs in this study, selection of the quality traits will be more efficient and accurate improvement of target traits will be fast achieved. Further, based on the information of genetic main effects of QTSs or interaction effects of QTSs with environments, it becomes possible for us to design an universal selection strategy effective for all the environments or a specific selection strategy for individual environments.

According to the association analysis, two QTSs (rs1610021 and rs1289107) of the four QTSs, which are involved in the genetic variation of three quality traits of rice, are located in the region of the known genes (Os06g0130100 and Os06g0124300), and the other two, rs1644460 and rs919289, lie in the upstream or the downstream of known genes (Os06g0130800 and Os07g0116900). Some of these genes have been well defined; for example, there is one QTS, rs919289, near the gene of Os07g0116900 that is described as NADH ubiquinone oxidoreductase, 20 kDa subunit domain containing protein. NADH plays essential roles in metabolism, which emerges as an adenine nucleotide that can be released from cells spontaneously and by regulated mechanisms [41, 42]. But the function of the others or the relationship between the gene and the three quality traits of rice still remains unexplored, such as Os06g0130100 whose annotation is similar to ERECTA-like kinase 1. It has been previously reported that ERECTA-family receptor-like kinases (RLKs) are redundant receptors that relate cell proliferation to organ growth and patterning [43]. Further investigation is needed to explain the association between RLK ERECTA and the three quality traits of rice.

Although the three rice quality traits exhibit different genetic architecture in the pattern of genetic effects, we believe that all of these are crucial genetic resources for improvement of the traits by selection of genetic effects. However, more validation is needed if these QTSs will be extended to other populations with different genetic background. In addition, more detailed whole-genome scanning, more powerful bioinformatics tools, and larger size of mapping populations are required since only causative polymorphisms with large effects can be detected given the size of the used RIL population [44].

## Figures and Tables

**Table 1:** Estimates of SNP additive effects and interaction effects with the environments under three different population structures (70%).

Chr	SNP ID	Pop	*a *		*ae*_1_		*ae*_2_		Power
			Par.	Est.	Par.	Est.	Par.	Est.	
1	28	100	−3.24	−3.32	2.65	2.67	−2.65	−2.46	99.00
		150		−3.39		2.81		−2.51	100.00
		200		−3.42		2.81		−2.45	100.00

2	100	100	−2.65	−2.79	4.05	4.16	−4.05	−3.79	100.00
		150		−2.90		4.26		−3.75	100.00
		200		−2.94		4.34		−3.73	100.00

3	93	100	−1.77	−1.86	3.24	3.34	−3.24	−3.17	87.00
		150		−1.84		3.34		−3.14	98.50
		200		−1.90		3.34		−3.09	100.00

Chr: the ordinal number for simulated chromosome; Pop: the population size; Power: the percentage of the detected SNP with significant effect at 0.05 levels; Par.: the true value of parameter in simulations; Est.: the estimate of parameter; *a*: additive effect; *ae*_1_ and *ae*_2_: the interaction effect of additive with environment 1 and 2, respectively.

**Table 2:** Estimates of epistasis and interaction effects with the environments under three different population structures (70%).

Chr.*i *	SNPID.*i *	Chr.*j *	SNPID.*j *	Pop	*aa *		*aae*_1_		*aae*_2_		Power
					Par.	Est.	Par.	Est.	Par.	Est.	
2	44	3	63	100	3.86	3.42	4.47	4.87	−4.47	−3.99	100.00
				150		3.24		5.05		−3.90	100.00
				200		3.21		5.12		−3.79	99.50

*aa*: additive-additive epistasis effect; *aae*_1_ and *aae*_2_: the environment-specific additive-additive epistasis effect; Pop, Power, Par., and Est. have same definitions as those in Table 1.

**Table 3:** Estimates of SNP additive effects and interaction effects with the environments under two different heritabilities.

Chr	SNPID	*a *			*ae*_1_			*ae*_2_			Power	
		Par.	Est.		Par.	Est.		Par.	Est.			
			I	II		I	II		I	II	I	II
1	28	−0.79	−0.81	−0.83	0.63	0.65	0.67	−0.63	−0.60	−0.58	100.00	100.00
2	100	−0.67	−0.67	−0.68	0.39	0.38	0.39	−0.39	−0.37	−0.37	100.00	100.00
3	93	−0.40	−0.41	−0.41	0.32	0.32	0.32	−0.32	−0.31	−0.31	69.00	70.00

I and II stand for two different heritabilities of 50% and 70%, respectively, which are the proportions of total phenotypic variation ascribed to SNP additive effects and additive-environment interaction effects; Power, Par., Est., *a*, *ae*_1_, and *ae*_2_ have the same definitions as those in Table 1.

**Table 4:** Estimates of epistasis and interaction effects with the environments under two different heritabilities.

Chr.*i *	SNPID.*i *	Chr.*j *	SNPID.*j *	*aa *			*aae*_1_			*aae*_2_			Power	
				Par.	Est.		Par.	Est.		Par.	Est.			
					I	II		I	II		I	II	I	II
2	44	3	63	0.39	0.38	0.38	0.17	0.18	0.17	−0.17	−0.18	−0.16	100.00	99.50

Power, Par., and Est. have the same definitions as those in Table 1; *aa*, *aae*_1_, and *aae*_2_ have the same definitions as those in Table 2.

**Table 5:** Detected SNPs with significant genetic effects.

Trait	QTS	Chr.	Allele	Effect	Predict	−log_10_⁡(*P*)	*h* ^2^ (*%*)	Total
AC	rs1610021	6	G/A	*a *	−2.20	26.94	16.89	68.67
	rs1644460	6	C/T	*a *	−3.85	79.97	51.78	

GC	rs1644460	6	C/T	*a *	15.02	61.32	52.15	58.58
				*ae*_1_	−3.48	2.52	2.80	
				*ae*_2_	3.47	2.11	2.80	
	rs919289	7	C/G	*a *	−3.96	4.97	3.63	

GT	rs1289107	6	G/A	*a *	−0.54	25.06	24.00	52.01
				*ae*_1_	0.25	3.49	5.00	
				*ae*_2_	−0.25	2.99	5.00	
	rs1644460	6	C/T	*a *	−0.35	10.73	9.81	
				*ae*_1_	−0.30	5.01	7.40	
				*ae*_2_	0.30	4.05	7.40	
	rs1289107 and rs1644460	6	G/A	*aa *	0.21	4.33	3.60	
				*aae*_1_	−0.16	1.76	2.20	
		6	C/T	*aae*_2_	0.17	1.61	2.20	

AC: amylose content; GC: gel consistency; GT: gelatinization temperature; *e*_1_: environment 1; *e*_2_: environment 2; *a*: additive effect; *ae*_1_ and *ae*_2_: environment-specific additive effect; *aae*_1_ and *aae*_2_: environment-specific additive-additive epistasis effect. −log_10_⁡(*P*) = −log_10_⁡(*P*-value). *h* ^2^ (%) = heritability (%) due to the genetic component effect.
